# Time-Resolved Structural Insight into Zinc Metal Plating in Aqueous Batteries

**DOI:** 10.1021/acs.chemmater.5c02962

**Published:** 2026-07-20

**Authors:** Lacey Roberts, Claire Ely, Elizabeth Allan-Cole, Julian Mars, Thomas Chaney, Denis Keane, Saul Lapidus, Erik Sarnello, Nicholas Weadock, Samuel Marks, Michael Toney

**Affiliations:** † Department of Chemical and Biological Engineering, 1877 University of Colorado Boulder, Boulder, Colorado 80303, United States; ‡ Materials Science and Engineering, 28259 University of Colorado, Boulder, Colorado 80303, United States; § Department of Materials Science and Engineering, Northwestern University, Evanston, Illinois 60208, United States; ∥ Northwestern Synchrotron Research Center, Argonne, Illinois 60439, United States; ⊥ X-ray Science Division, Advanced Photon Source, Argonne National Laboratory, Argonne, Illinois 60439, United States; # Renewable and Sustainable Energy Institute (RASEI), University of Colorado at Boulder, Boulder, Colorado 80303, United States

## Abstract

Zinc metal anodes are promising for next-generation aqueous batteries due to their low electrochemical potential, high volumetric capacity, and global abundance. However, their application is hindered by irreversibility caused by parasitic reactions on the anode, including hydrogen evolution, dendrite formation, and zinc hydroxide sulfate (ZHS) buildup. The structural origins of electrochemical irreversibility in aqueous zinc cells are related to these parasitic side reactions, plating/stripping efficiency, and the crystallographic evolution of zinc anodes during cycling. Here, we use *operando* X-ray diffraction to track zinc plating and stripping under varying current densities and capacities, uncovering key structural changes that govern reversibility. Zinc plates with a preferred (002) orientation during the first cycle; however, subsequent cycling disrupts this texture, especially at high capacities, shifting the orientation toward Zn(100) or producing random orientations. We show that ZHS formation is governed primarily by the total time spent at low potentials rather than current density or cycle number, implicating calendar aging as a dominant degradation pathway. Dead zinc and ZHS formation both correlate with reduced Coulombic efficiency, but dead zinc appears to be the dominant factor in early cycle reversibility loss. These findings provide structural insight into degradation mechanisms and highlight the importance of engineering interfaces and electrolytes to control crystallographic orientation and parasitic phase formation.

## Graphical abstract

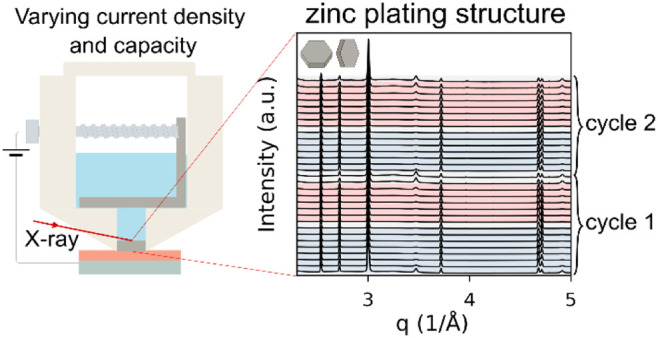

## Introduction

The solution to the global climate crisis should be thoughtful and multifaceted. One aspect of this solution includes creating a resilient, just, and fossil-free energy infrastructure that includes batteries as one of several energy storage solutions, for example, for electric vehicles and grid storage. Commercially mature lithium-ion batteries are now widely used, but safety, sustainability, energy and environmental justice, global supply chain, local manufacturing, and cost concerns leave room for improvement that can be met by alternative battery technologies. In particular, the flammability of lithium-ion batteries remains a significant concern, as indicated by reports of consumer device failures caused by battery-related fires.^1,2^ These dangers are magnified at the grid scale, as demonstrated by the recent fire at the Moss Landing energy storage facility in California. This incident led to the evacuation of over 1,000 residents and the detection of heavy metals in dust several miles from the site.^3,4^ Supply chain resilience is also critical, as disruptions to the mining or refining of key battery materials like lithium, cobalt, and nickel can threaten clean energy deployment timelines and national energy security.^5^

Alternative battery chemistries are required to meet the specific demands for different applications, to improve safety, and to diversify the supply chain.^6^ For instance, electric vehicles require high gravimetric energy density, making lithium-ion batteries well-suited for this application. However, sodium-ion chemistries have the potential to be competitive and can be manufactured locally.^7^ Conversely, for grid-scale energy storage, cost, safety, and longevity are primary considerations.^8^ Here, zinc metal batteries (ZMBs) offer potential advantages such as low cost, high volumetric specific capacity (5854 mA/cm^3^), and abundant zinc supply.^9^ A unique advantage of ZMBs is their compatibility with aqueous, nonflammable electrolytes, due to zinc’s low reduction potential (−0.76 V vs the Standard Hydrogen Electrode). Additionally, zinc has widespread global abundance, with more than 700,000 t of zinc mined in the U.S. each year.^9,10^ The abundance of ZMB materials alleviates societal and ethical challenges associated with rare-earth metal and cobalt mining and diversifies the supply chain.

At the zinc metal anode in a ZMB, zinc is plated by reducing Zn^2+^ to Zn^0^ during charging and stripped by oxidizing Zn^0^ to Zn^2+^ upon discharge. However, the reversibility of these processes is limited, particularly over extended cycling, which contributes to poor long-term stability and short calendar life of ZMBs.^11,12^ This irreversibility is primarily attributed to parasitic side reactions, including hydrogen evolution, the formation of electrically isolated (“dead”) zinc, and dendrite formation.^13^ Dendrites not only cause short circuiting, but exacerbate corrosion reactions by increasing the zinc metal active area exposed to electrolyte.^14^ However, we do not know the efficiency of zinc metal plating and stripping, and quantitatively how much charge goes to plating/stripping vs parasitic side reactions.

A ubiquitous corrosion byproduct at ZMB anodes is zinc hydroxide sulfate (ZHS), Zn_4_(OH)_6_SO_4_·xH_2_O^15^. This flake-like, layered structure forms in mildly acidic aqueous zinc batteries when hydrogen has evolved. Hydrogen can evolve from zinc dissolution (eq 1), or electrochemically (eq 2).

1 $$Zn^{0}+2H_{2}O\to Zn^{2+}+2OH^{-}+H_{2}$$

2 $$H_{2}O+2e^{-}\to 2OH^{-}+H_{2}$$

Both pathways result in a local alkaline pH near the zinc anode. The OH^–^ anions subsequently react with local Zn^2+^ and SO_4_^2–^ ions and H_2_O, forming ZHS (eq 3).

3 $$4Zn^{2+}+6OH^{-}+SO_{4}^{2-}+\gamma H_{2}O\to Zn_{4}SO_{4}{\left(OH\right)}_{6}\cdot \gamma H_{2}O$$

ZHS has also been shown to form on zinc foil soaked in 1 M ZnSO_4_ without an applied bias, suggesting its formation by chemical reaction is thermodynamically favored.^12,16,17^ While ZHS has been observed to form on the anode and cathode of ZMBs, its growth behavior and relationship to cycling parameters remain poorly understood, which is primarily due to the lack of *operando* data quantifying ZHS formation and subsequent evolution.

Another critical factor influencing zinc metal battery performance is the crystallographic structure and texture of the plated zinc. Zinc deposits in a hexagonal close-packed (HCP) structure and exhibits a strong tendency to electrodeposit as hexagonal platelets, where zinc atoms condense onto the (100) facet, resulting in a platelet that maximizes the (002) surface area (Figure 1b–d).^18^ These platelets can be oriented with different crystallographic planes parallel to the substrate surface. For example, zinc plating with the (002) plane parallel to the substrate (Figure 1c) exhibits distinct morphologies and behaviors compared to zinc plating with the (100) plane parallel to the substrate (Figure 1d).

**Figure 1:**
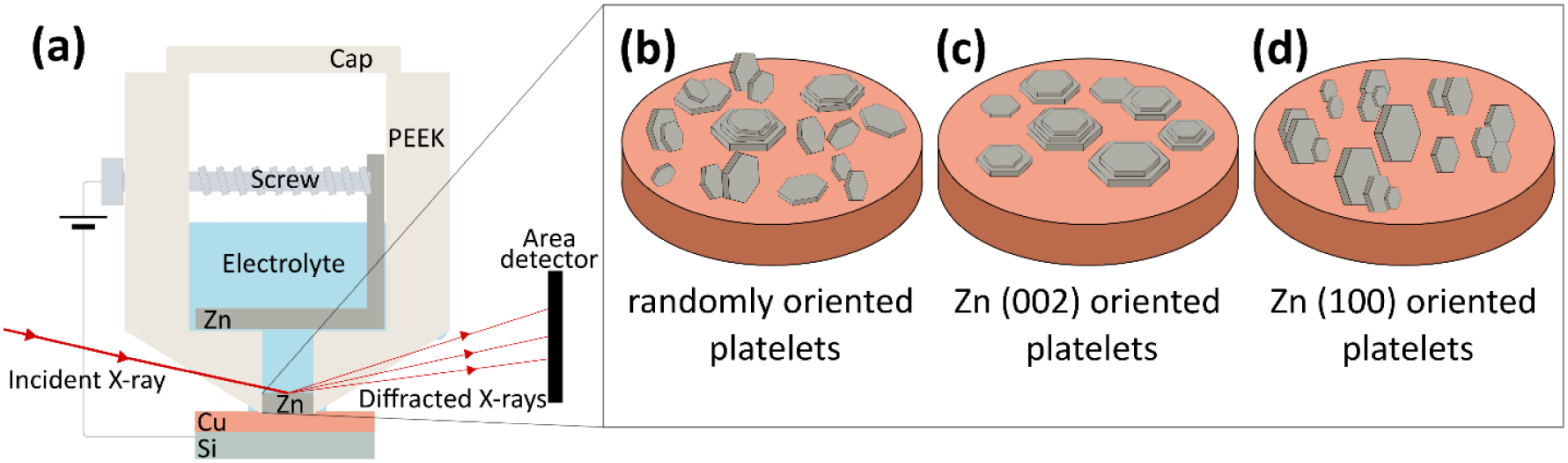
a) Cone cell constructed of polyether ether ketone (PEEK), where the working electrode is the copper substrate deposited on a silicon wafer, the reference and counter electrodes is the zinc foil connected to the circuit with a stainless-steel screw, and the electrolyte is aqueous 1 M ZnSO_4_. The incident X-rays penetrate through the PEEK and the diffracted X-rays are collected at the area detector. Schematic of zinc plated onto a copper substrate with (b) random, (c) Zn(002) or (d) Zn(100) oriented parallel to the substrate.

Prior research has often focused on promoting growth with the (002) plane parallel to the substrate to form compact platelet structures.^18–22^ Compact platelets are advantageous as they can suppress dendrite formation and enable smoother, oriented growth with the substrate, contributing to more stable cycling. However, in recent work, the use of a monocrystalline (100) faceted zinc anode resulted in the highest overall reversibility, despite the conventional preference for the (002) orientation.^23^ These uncertainties highlight the importance of understanding and controlling zinc crystallographic orientation as a key strategy for enhancing the reversibility and long-term stability of ZMBs.

Cycling parameters such as current density and cycled cutoff capacity play a significant role in determining zinc crystallography, morphology, and ultimately, battery performance. Contrary to lithium-ion batteries, where higher current densities are often detrimental to performance, in zinc metal anodes, higher current densities lead to more compact and densely packed zinc deposits that improve cyclability.^24–26^ Additionally, increasing the plating capacity results in the formation of larger zinc platelets.^25^ However, the dynamic evolution of zinc structure and parasitic phase formation under different current densities and capacities has not been systematically explored using *operando* techniques.

While optimizing current density and cutoff capacity can improve zinc morphology and performance, long-term stability required for the commercialization of ZMBs remains a challenge. Efforts to mitigate these challenges in ZMBs have included coating the zinc metal with an artificial solid electrolyte interface layer, engineering the zinc metal anode geometry, and introducing electrolyte additives and cosolvents.^27–30^ These approaches aim to reduce water activity in the bulk and near the zinc electrode, resulting in less hydrogen evolution, fewer parasitic losses, and a smoother zinc deposition. For example, water-in-salt (WIS) electrolytes use a high concentration of salt to minimize the total amount of water present.^31,32^ While effective at increasing battery lifetime and Coulombic efficiency, WIS electrolytes are viscous and expensive due to the high volume of salt needed.^33^

Another strategy for improving ZMB reversibility is the addition of organic additives to the aqueous ZnSO_4_. Some of these additives are hypothesized to change the bulk solvation structure of Zn^2+^ in the electrolyte, while others are hypothesized to adsorb to the zinc metal surface.^34–37^ Adsorption of additives on the zinc electrode surface will subsequently exclude water from the interface, acting as a barrier to water molecules and therefore preventing hydrogen evolution and corrosion reactions, but still allowing Zn^2+^ mass transfer.^38,39^

Here, we will focus on the ZnSO_4_ electrolyte, which is the most common ZMB electrolyte salt. To inform these ZMB improvement strategies, this study aims to elucidate the extent and causes of irreversibility of zinc metal anodes in ZnSO_4_, thereby improving our understanding of the plating and stripping mechanisms and capacity loss processes, which can inform the design of engineered electrolytes. To investigate these degradation mechanisms in zinc metal anodes, we employ quantitative *operando* XRD, a technique that is well-suited for characterizing structural aspects of electrochemical plating processes.^40^ By correlating diffraction peak intensities with electrochemistry during cycling, we quantitatively assess how different current densities and capacities influence zinc deposition efficiency and long-term reversibility. Our findings provide information on the interplay between Zn(002) and Zn(100) preferred orientations, the emergence of dead zinc, and the timeline of ZHS accumulation, offering a more comprehensive view of the structural changes that contribute to performance degradation.

## Experimental Methods

A type of beaker cell, the conecell,^41–45^ was used to enable *operando* XRD measurements. The conecell (Figure 1a, S1) consists of a 0.1 mm thick zinc foil (MTI) as a counter and reference electrode, a 1 molal (m) zinc sulfate electrolyte (prepared from ZnSO_4_·H_2_O, ≥99.9%, Sigma-Aldrich), and a copper substrate as a working electrode. Figure 1a shows a cross-section schematic of the conecell and its components with the incident and diffracted X-ray directions shown. The substrate was prepared via thermal evaporation of a 100 nm thick copper layer on a 500 μm thick (100) silicon wafer with a 5 nm chromium adhesion layer between the silicon and the copper to promote the adhesion of the copper film. The resulting copper film has a preferential (111) orientation (Figure S2). The resulting smooth copper surface allows for the precise connection of the conecell without leaking. The conecell was constructed out of polyether ether ketone (PEEK) for chemical inertness and minimal X-ray absorption.

The cells were cycled at constant current densities of 2.5, 5, 10, or 15 mA/cm^2^. Zinc plating was performed to fixed areal capacities of 2, 2.5, or 10 mAh/cm^2^, while zinc stripping was terminated at a voltage cutoff of 0.5 V vs Zn, corresponding to the onset of a sharp voltage increase associated with depletion of electrochemically active zinc. An open current is applied for 10 min between each plating and stripping step. Coulombic efficiency (CE) was calculated as the ratio of total charge stripped to total charge plated, expressed as a percentage. The CE values reported represent cumulative efficiency to enable direct comparison with degradation products such as dead zinc and ZHS, which accumulate over multiple cycles.

*Operando* XRD experiments were conducted at the Advanced Photon Source 11-BM-B at 27 keV with a beam size of 1 × 1 mm and a 645 mm detector distance. The X-ray beam was aligned to the working electrode, where zinc was plated and stripped onto the copper substrate. Measurements were done at incidence angles of 5°, 8°, and 12° and with a 2-s exposure time using a PerkinElmer area detector. To capture initial plating behavior, a 12° incidence angle was used to collect XRD data. After 3 min of measurements at 12°, XRD data were taken every 2 s cycling between 2°, 5°, and 8° incidence angles. After 10 min of open current hold, this same process was used for the second cycle. The varying incidence angles were used to isolate X-ray absorption effects.

The resulting data are detector images, which were converted to q-space and integrated from χ = 10° to 170° into I vs q profiles, as shown in Figure 2a. Here, q is the scattering vector analogous to the 2Θ angle typically reported for laboratory XRD measurements. Each diffraction peak of interest was fitted to a Gaussian curve with area, position, and full width at half-maximum (fwhm) as fitting parameters using the Python lmfit package (Figure S3). When comparing data collected at different incident angles, the missing wedge was accounted for during integration.^46,47^ However, higher incident angles resulted in increased shadowing of the low Qz region by the electrochemical cell, requiring exclusion of a larger portion of χ-space. To ensure a constant illuminated volume, detector geometry, and integration range throughout cycling, all quantitative analysis presented in this work therefore focuses on data collected at a fixed 5° incident angle. Python scripts and raw data can be found on GitHub (https://github.com/laro9334/Operando_XRD).

**Figure 2:**
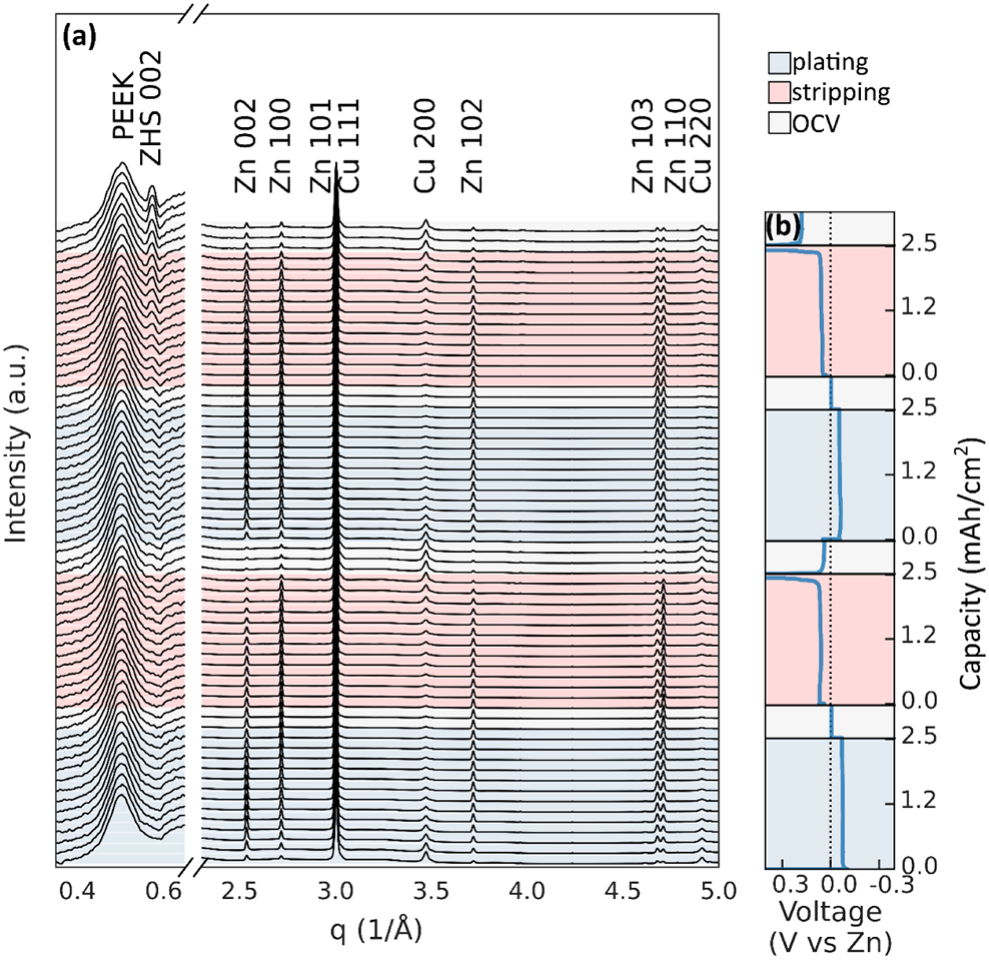
Cell with current density of 2.5 mAh/cm^2^ and a capacity of 2.5 mAh/cm^2^ a) X-ray diffraction patterns with selected diffraction peaks labeled and (b) corresponding voltage profile where the shaded blue regions indicate plating zinc, shaded gray indicates 10 min open circuit voltage with no capacity change, and shaded red indicated stripping zinc regions. Time increases from bottom upward.

## Results and Discussion

To examine the capacity and time-dependent structure and orientation of the deposited zinc and ZHS, X-ray diffraction (XRD) measurements were performed during two plating and stripping cycles. The results of the diffraction measurement are shown in Figure 2. Figure 2a shows *operando* XRD patterns collected during galvanostatic cycling. The diffracted intensity is plotted as a function of the scattering vector q, ranging from 0.4 to 5.0 Å^–1^, with each scan corresponding to a different time point during cycling. The background color shading denotes electrochemical states: plating (red), stripping (blue), and open-circuit voltage (gray). Several key reflections are labeled, including peaks corresponding to the PEEK cell, ZHS(002), Zn­(002,100,101,102,103,110), and Cu­(111,200,220). Figure 2b displays the corresponding voltage profile overlaid with shaded regions indicating the same electrochemical segments as in Figure 2a.

Figure 2a shows that the zinc intensity increases as the capacity increases and then decreases as the zinc is stripped, which is further quantified in Figure 3. In contrast, the copper peak intensity decreases during zinc plating due to the growing zinc layer, which attenuates the X-rays incident on the underlying copper substrate. The ZHS peak at 0.575 Å^–1^ increases continuously with total cycling time. Note the presence of zinc metal XRD peaks during the open circuit voltage (OCV) rest cycles (gray) after the stripping step; this shows the presence of “dead” zinc as explained below.

**Figure 3:**
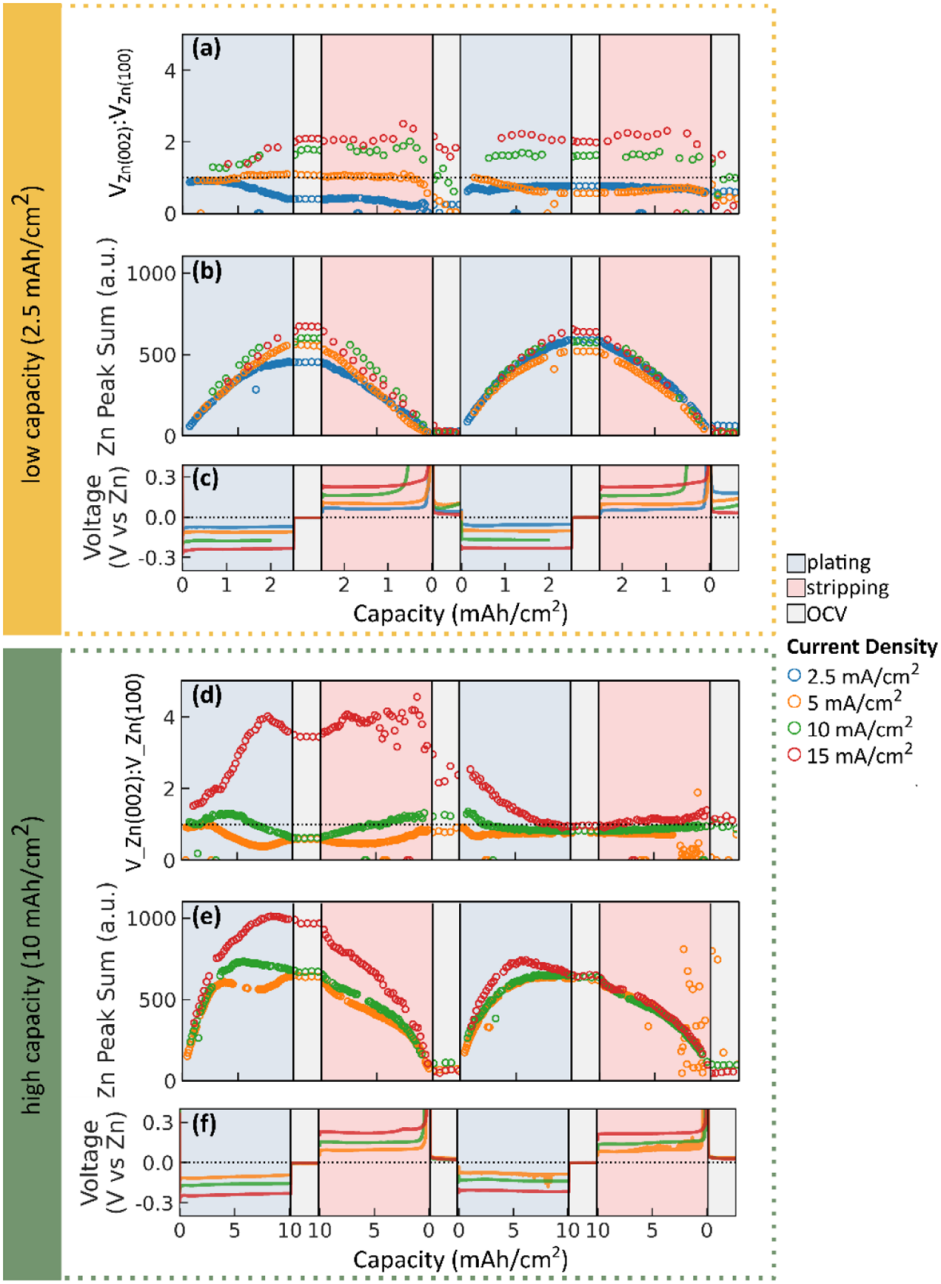
(a) Volume ratio of Zn(002) to Zn(100) from diffraction peak areas, (b) sum of the diffracted peak areas of Zn(002), Zn(100), and Zn(102), (c) voltage profiles for current densities 2.5, 5, 10, and 15 mA/cm^2^ and capacity of 2.5 mAh/cm^2^. (d) Volume ratio of Zn(002) to Zn(100) from diffracted peak areas, (e) sum of the diffracted peak areas of Zn(002), Zn(100), and Zn(102), (f) voltage profiles for current densities 5, 10, and 15 mA/cm^2^ and capacity of 10 mAh/cm^2^.

The integrated area under the diffraction peaks in Figure 2a quantifies the diffracting volume of zinc with the corresponding preferred orientation between χ = 10° to 170° (eqs 4,5). These integrated intensities are then compared with the electrochemical cycling rate and capacity (Figure 2b).

From the diffraction results, we examine the relative abundance of zinc platelets with Zn(002) and Zn(100) orientations (which are orthogonal directions). To quantify the amount of Zn(002)-oriented platelets relative to Zn(100), we used eq 4, where V_Zn002_/V_Zn100_ represents the volume ratio of diffracted Zn(002) to Zn(100) platelets.^48^ This ratio is calculated using the integrated peak area (I), the structure factor (F), and the Lorentzian factor (L). The Lorentzian factor, defined in eq 5, incorporates the multiplicity factor *m* and the Bragg diffraction angle 2θ.^48^ The resulting ratio gives us insight into how the plated zinc is oriented and how the orientation evolves with cycling.

4 $$\frac{V_{Zn002}}{V_{Zn100}}=\frac{I_{Zn002}}{I_{Zn100}}\times \frac{|F_{Zn100}{|}^{2}}{|F_{Zn002}{|}^{2}}\times \frac{L_{Zn100}}{L_{Zn002}}$$

5 $$L_{Znhkl}=\frac{m_{Znhkl}}{2\sin\left(\theta \right)\sin\left(2\theta \right)}$$

These results are shown in Figure 3a,d. A volume ratio of Zn(002):Zn(100) greater than 1 indicates preferential orientation of plated zinc with the Zn(002) plane oriented parallel to the substrate. Each data set is color-coded by current density (2.5–15 mA/cm^2^), and the voltage profiles (Figure 3c,f) for each current density and cutoff capacity reflect the increased overpotential associated with higher current densities (Figure S4).

During the first cycle plating for the lower and higher cutoff capacity (Figure 3a,d) cells, V_Zn(002):V_Zn(100) generally increases with current density, with the highest current densities consistently showing enhanced Zn(002) texture. High current densities increase the plating overpotential, resulting in the formation of many small nuclei.^49^ Densely distributed nuclei promote uniform lateral growth along the [100] direction, resulting in a plated zinc layer that maximizes the (002) surface area.

Comparing the two capacities, the effect of current density of plating orientation is even stronger during the first cycle of the 10 mAh/cm^2^ cells (Figure 3d) compared to the same current density cells at 2.5 mAh/cm^2^ (Figure 3a). Interestingly, the plating orientation is most strongly influenced by current density during the first cycle. During the second cycle, the zinc crystalline orientation becomes random (no preferred orientation) at high capacities (Figure 3d), while the low cutoff capacity cells with a high current density maintain the Zn(002) texture (Figure 3a). These results show that the stability of the current density-induced crystallographic texture depends on cutoff capacity. We speculate that this change could be due to loss mechanisms associated with higher capacities, such as hydrogen evolution and ZHS formation, which alter the local electrode environment by increasing pH and consuming Zn^2+^ near the surface. These changes shift the effective plating region and influence the initial nucleation pathway, leading to different zinc crystallographic textures.

To give insight into the effect of current density and capacity on zinc metal plating efficiency during cycling, we look at the initial slopes of zinc metal peak area vs capacity, which are proportional to the plating efficiency when X-ray absorption is small. Figure 3b,e shows the total diffracted zinc area as a function of capacity. The slopes of Figure 3b,e are proportional to zinc metal plating efficiency; larger slopes indicate that more zinc metal is plated with the same amount of charge passed.

At higher capacities, however, the interpretation of the diffraction intensity becomes more complex. As the zinc layer thickens, the diffracted intensity begins to plateau with increasing capacity due to Beer’s Law, as increased X-ray absorption limits the penetration of the beam into the film. In this regime, the X-rays no longer probe the full thickness of the Zn deposit, and the measured signal reflects a constant effective sampling volume rather than the total amount of plated zinc.

In addition to absorption, spatial heterogeneity in Zn deposition may further contribute to the differences in plateau behavior between cells. Because the X-ray beam samples only a finite region of the electrode, Zn deposited outside the illuminated region will not contribute to the measured diffraction intensity. One possible source of such heterogeneity is preferential Zn deposition near the perimeter of the electrode (“edge effects”). A simple geometric estimate (SI Section S1) shows that the beam samples disproportionally less area around the edge of a circular area. As lower current densities are more prone to heterogeneous Zn deposition, this effect may contribute to the lower apparent plateau intensities observed at lower current densities in Figure 3b,e.

Therefore, both absorption and nonuniform spatial deposition can lead to an underestimation of the total plated Zn at higher capacities. To minimize these effects, we restrict our analysis of plating efficiency to the low-capacity regime, where the zinc thickness is small, absorption is negligible, and the diffraction signal more directly reflects the total plated Zn.

The plating slopes for the first and second cycles are quantified and plotted as a function of current density in Figure 4a,c, with the corresponding fits provided in Figures S5–11. The uncertainties shown in Figure 4 are provided in the SI and are propagated in the dead Zn fraction analysis (Table S2). For the first cycle, the plating slope shows little dependence on current density, particularly at a high cutoff capacity. In contrast, during the second cycle, an increasing current density results in decreased slopes for the low cutoff capacity cells (Figure 4a), whereas the high cutoff capacity cells (Figure 4c) exhibit a minimal dependence on current density. Notably, the high cutoff capacity cells also display a pronounced reduction in plating slope from the first to the second cycle. This trend suggests that at a high cutoff capacity, the electrode surface undergoes significant morphological or chemical changes during the first plating step, such as roughening, dead zinc accumulation, or parasitic product formation, which promote increased hydrogen evolution reaction (HER) during the second cycle. The increased HER competes with zinc deposition for the applied current, reducing the efficiency of zinc plating and thereby lowering the plating slopes in the second cycle.

**Figure 4:**
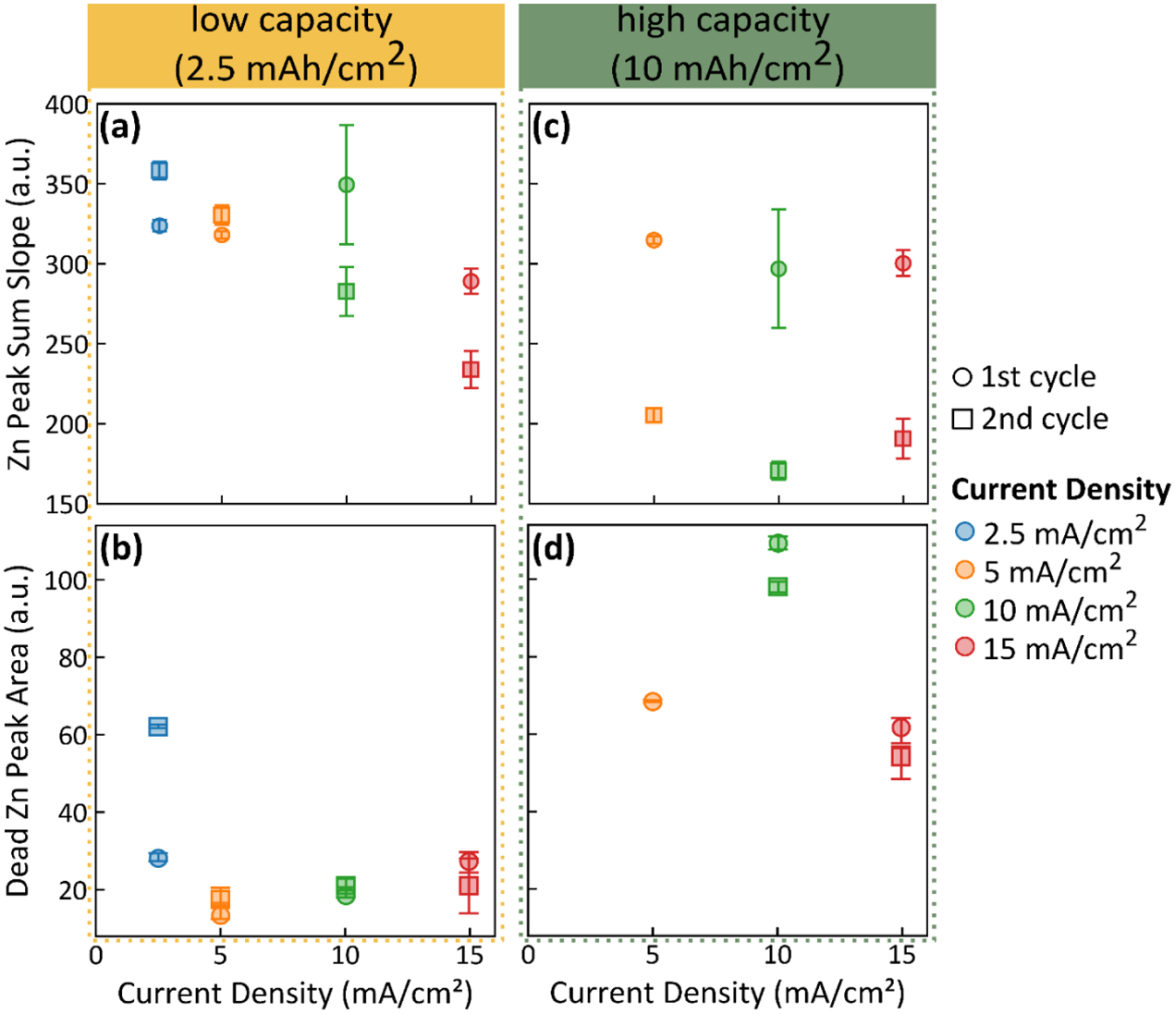
(a) Rate of change of the sum of diffracting volumes of Zn(002), Zn(100) and Zn(102) vs capacity vs current density, (b) median sum of diffracted zinc peak area in dead zinc regime for 2.5, 5, 10, and 15 mA/cm^2^ and capacity of 2.5 mAh/cm^2^. (c) Rate of change of the sum of diffracting volumes of Zn(002), Zn(100) and Zn(102) vs capacity vs current density, (d) median sum of diffracted zinc peak area in dead zinc regime for 5, 10, and 15 mA/cm^2^ and capacity of 10 mAh/cm^2^.

While zinc plating efficiency offers insight into how effectively charge translates to active material, further understanding of reversibility requires examining the residual zinc that remains after stripping, commonly referred to as “dead” zinc. Dead zinc refers to zinc metal that has lost contact with the electrode and therefore does not participate in any further stripping or plating cycles. In this study, we operationally define dead zinc as any residual, metallic zinc diffraction signal that persists after the cell reaches the cutoff potential of +0.5 V vs Zn during stripping, recognizing that this signal may include both electronically isolated Zn and Zn that is kinetically inaccessible under the stripping conditions used here. In Figure 3, the blue and red shaded regions indicate plating and stripping periods, respectively, while the gray regions denote open-circuit potential (OCV) holds. Because the stripping step is terminated once the voltage reaches 0.5 V vs Zn, any residual zinc diffraction signal observed during the subsequent OCV period (the second and fourth OCV gray regions) is due to the presence of “dead” zinc that was not removed during the electrochemical stripping process.

Figure 3b,e illustrates that the diffracted zinc metal signal is nonzero after the first stripping cycle, showing that dead zinc reduces the Coulombic efficiency even at low cycle numbers. We further quantify this by taking the median diffraction signal over the 10 min OCV and plot this vs current density for the first and second cycles. While there is little correlation in the amount of dead zinc with current density (Figure 4b,d), the higher cutoff capacity cells (Figure 4d) show more dead zinc. This is likely due to more zinc being plated in total, providing more opportunity for dead zinc to form. The scaling of dead zinc with cutoff capacity but not with current density suggests that the dominant factor is the total zinc mass deposited rather than the plating rate. At higher capacities, larger deposits may promote structural inhomogeneities, such as disconnected structures or porous morphologies, which are more prone to electrical isolation during stripping and thus contribute to dead zinc formation.

Additionally, the amount of dead zinc remains relatively constant during the OCV time periods (Figure 3b,e), indicating that there is minimal dissolution of dead zinc over short rest durations. While other studies have shown dramatic zinc dissolution during OCV, the 10 min of rest used in this study was not sufficient to observe this.^12^

We can estimate the quantitative amount of dead zinc from the ratio of the dead zinc peak areas in Figure 4b,d to that of the total plated zinc extrapolated from the initial slopes (Figure 4a,c) times the plated capacity. This shows that the amount of dead zinc is ≈2–7% of the total plated zinc for both cutoff capacities after the first and second cycles (SI Section S2). The relatively constant fraction across current densities is consistent with the hypothesis that dead zinc scales with total zinc deposited rather than plating rate.

While dead zinc reflects one form of irreversible loss stemming from the physical disconnection of plated metal, another critical contributor to reduced reversibility is the formation of parasitic byproducts. Zinc hydroxide sulfate (ZHS) is a common parasitic reaction product that also serves as an indicator of hydrogen production. The insulating ZHS will deplete active zinc ions (loss of zinc inventory) and partially passivate the zinc anode.^15^ Figure 5a,c shows the peak area of ZHS(002) with cycling, with the corresponding voltage profiles in Figure 3c,f. ZHS begins to form primarily during the first cycle stripping step, with only partial or no reversibility observed during the subsequent plating cycle. Notably, ZHS formation is more pronounced at lower current densities across both cutoff capacity conditions. However, prior work has shown that ZHS can form even in the absence of an applied bias,^16,17^ suggesting calendar time may contribute to its accumulation. To disentangle these effects, we next investigate the relationship between ZHS formation and the total cycling time of the cells.

**Figure 5:**
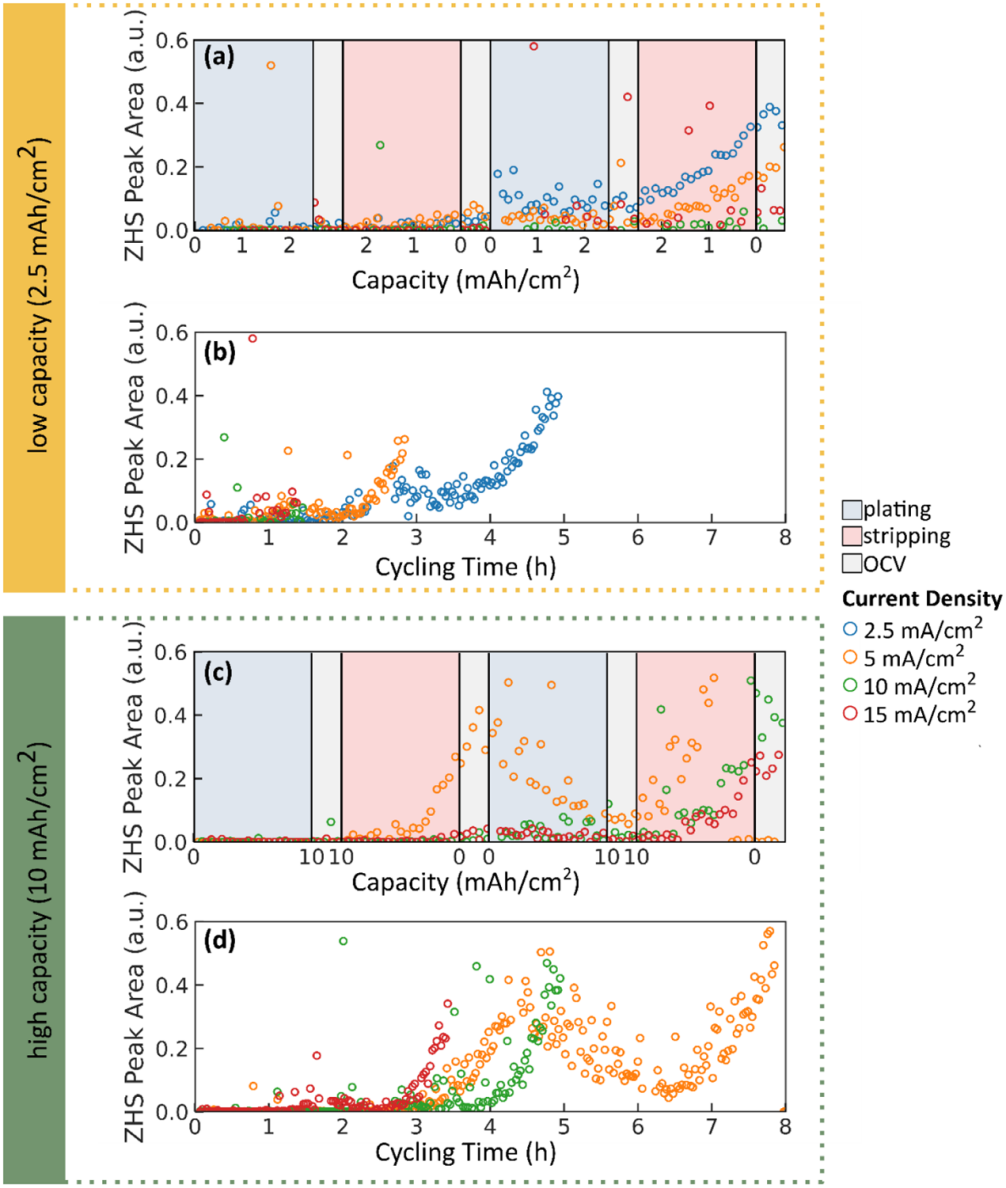
(a) ZHS(002) peak area vs capacity and (b) cycling time for 2.5, 5, 10, and 15 mA/cm^2^ and capacity of 2.5 mAh/cm^2^. ZHS(002) peak area vs (c) capacity, and (d) cycling time for 5, 10, and 15 mA/cm^2^ and capacity of 10 mAh/cm^2^.

Figure 5b,d shows the area of ZHS(002) vs total cycling time, where time equals to zero (h) is when the negative current is applied for the first plating cycle. As such, the lower current densities and higher cutoff capacities result in longer cycling times. Figure 4b,d demonstrates that across the different cells tested, the ZHS formed 2–3 h after cycling begins, independent of current density and cycle number. A higher current density results in less ZHS at a given cycling capacity; however, the shorter cycling time obscures the fact that the total ZHS accumulation is similar when accounting for the duration of cycling.

Notably, one cell (5 mA/cm^2^ current density with a 10 mAh/cm^2^ cutoff capacity) shows a decrease in ZHS intensity during the second plating cycle. We hypothesize that this occurs because these are the only conditions under which ZHS was already present before the second cycle, suggesting that ZHS formation is either (1) partially reversible during zinc plating, or (2) localized on the substrate surface such that subsequent zinc deposition obscures its signal.

Regardless of cycling conditions, ZHS continues to build up over time. This strongly implicates time at low potentials (e.g., between ≈−0.2 and ≈0.2 vs Zn/Zn+) as the determining variable in ZHS formation. Therefore, we suggest the ZMB community be cautious when attributing poor cycling performance solely to lower current densities and to consider calendar lifetime when evaluating cell improvements.^50^

As both dead zinc and ZHS formation reduce the amount of active zinc available for cycling, we next examine their relationship with CE to understand the overall degradation landscape better. Figure 6a–d display the amounts of dead zinc and ZHS after the first and second stripping steps, plotted against CE. Across varying current densities and cutoff capacities, dead zinc and ZHS amounts generally increase as CE decreases, especially at low cell cutoff capacities. While we cannot precisely quantify the relative contribution of ZHS formation and dead zinc to the decline in CE, we hypothesize that the formation of dead zinc is the dominant factor. This is supported by the observation that ZHS accumulation correlates more strongly with total cycling time rather than with the total charge passed (Figure 5b,d).

**Figure 6:**
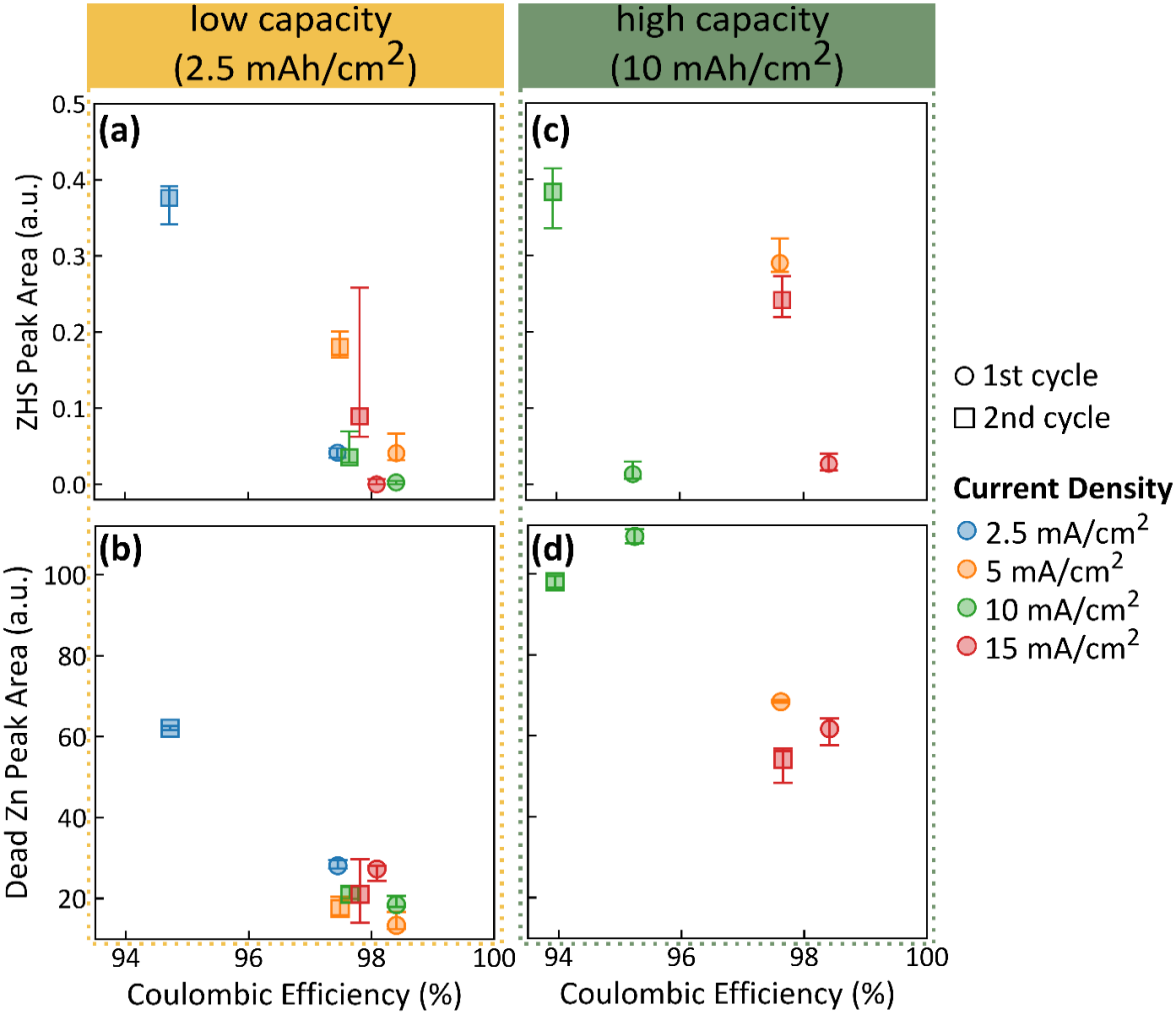
(a) Dead zinc peak area and (b) ZHS peak area after the first (circle) and second (square) cycles vs Coulombic efficiency, for cells with current density 2.5, 5, 10, and 15 mA/cm² and capacity of 2.5 mAh/cm^2^. (c) Dead zinc peak area and (d) ZHS peak area after the first (circle) and second (square) cycles vs Coulombic efficiency, for cells with current density 5, 10, and 15 mA/cm² and capacity of 10 mAh/cm^2^.

In conclusion, this work reveals how zinc crystallographic orientation, plating efficiency, and parasitic phase formation evolve dynamically during electrochemical cycling. Using *operando* XRD, we show that Zn(002) texture is favored during initial plating at high current densities but the Zn(002) texture is lost with continued cycling, especially at high cutoff capacities. This loss of textural control coincides with increased dead zinc formation and reduced plating efficiency. Additionally, ZHS formation is strongly linked to time spent at low potentials, independent of current density, emphasizing the role of calendar aging in zinc anode degradation. While dead zinc appears to be the primary driver of early CE losses, persistent ZHS buildup contributes to long-term passivation. These findings suggest that effective electrolyte and interface design strategies should (i) suppress time-dependent ZHS formation and (ii) promote compact Zn deposition to minimize dead Zn formation during stripping. Future operando studies with electrolyte additives will be key to further improving the reversibility and longevity of aqueous zinc metal batteries.

## Supplementary Material



## Notes

Variables used for quantifying Zn(002):Zn(100); photograph of conecell; full detector image; XRD peak fit example; plating, stripping, and OCV potentials; slopes; geometric estimate of X-ray sampling of edge enriched regions; estimation of dead Zn fraction from diffraction peak area; SEM of plated zinc (PDF)
