# Supplementary material for: Time-Resolved Structural Insight into Zinc Metal Plating in Aqueous Batteries
Source: Chem Mater. 2026 Jul 20;38(15):7549–58. doi: 10.1021/acs.chemmater.5c02962 (PMC13471730; doi:10.1021/acs.chemmater.5c02962)
Supplement: Supplementary file 1 [file cm5c02962_si_001.pdf]

Supplementary Information

Time-Resolved Structural Insight into Zinc Metal Plating in Aqueous Batteries

Lacey Roberts[1], Claire Ely[1], Elizabeth Allan-Cole[1], Julian Mars[2], Thomas Chaney[2], Denis Keane[3,4], Saul Lapidus[5], Erik Sarnello[5], Nicholas Weadock[2], Samuel Marks[2*], Michael Toney[1,2,6]*

1. Department of Chemical and Biological Engineering, University of Colorado Boulder, Boulder, CO 80303, USA
2. Materials Science and Engineering, University of Colorado, Boulder, CO 80303, USA
3. Department of Materials Science and Engineering, Northwestern University, Evanston, Illinois 60208, USA
4. Northwestern Synchrotron Research Center, Argonne, IL 60439, USA
5. X-ray Science Division, Advanced Photon Source, Argonne National Laboratory, Argonne, Illinois 60439, USA
6. Renewable and Sustainable Energy Institute (RASEI), University of Colorado at Boulder, Boulder, CO 80303, USA

* Email: michael.toney@colorado.edu, Samuel.Marks-1@colorado.edu

This supplementary information contains tables and figures to complement the main text.

Table S1. *Values used for comparing diffracted intensities from Equation 4 and Equation 5 in main text.*

Compound	hkl	2θ	ν_a (Å^3)	F_T	*m*
Zn	002	10.81	28.85	48.7591	2
Zn	100	11.64	28.85	23.8166	6
Zn	102	15.91	28.85	20.969	12

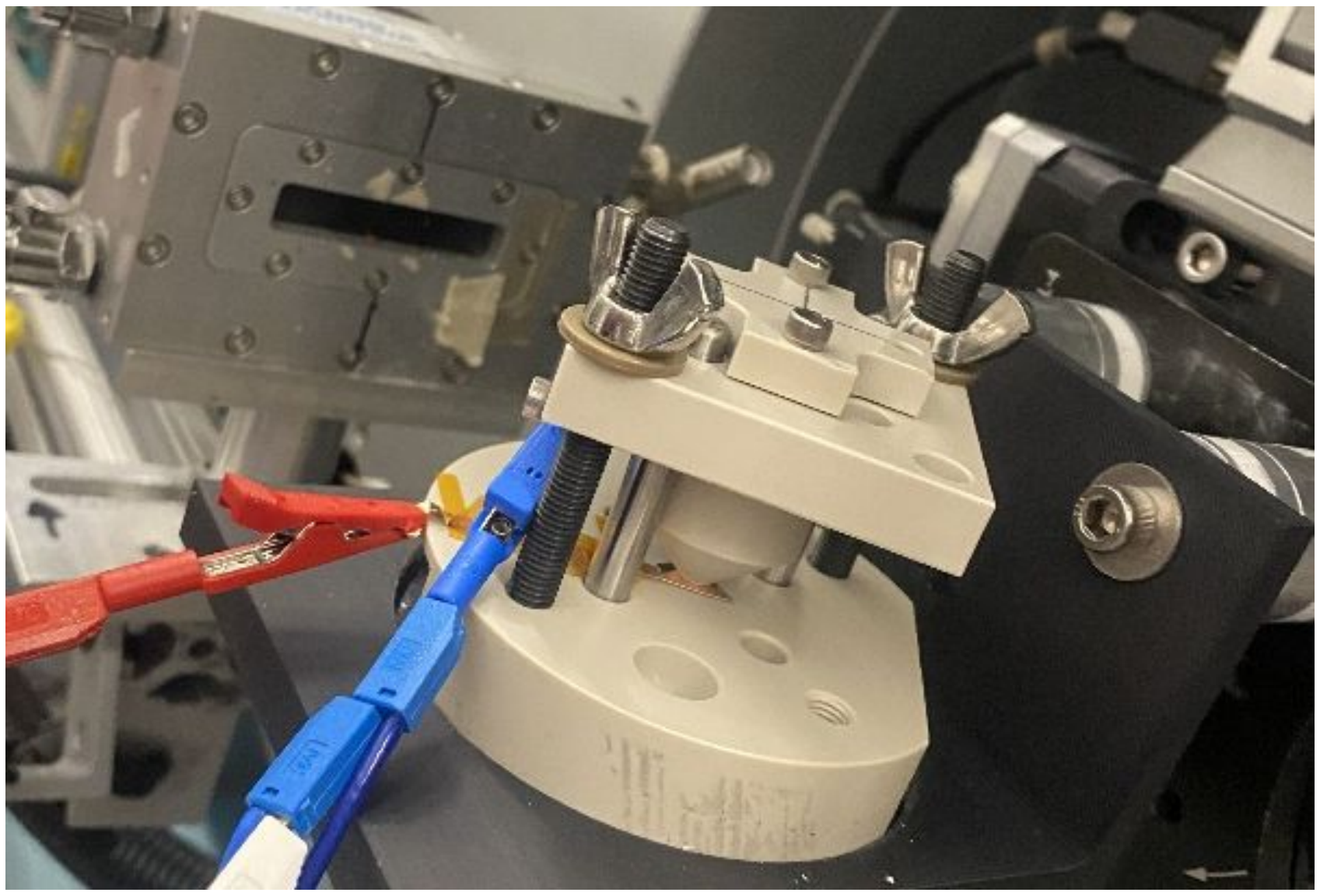

Figure S1. Conecell on beamline with potentiostat connections.

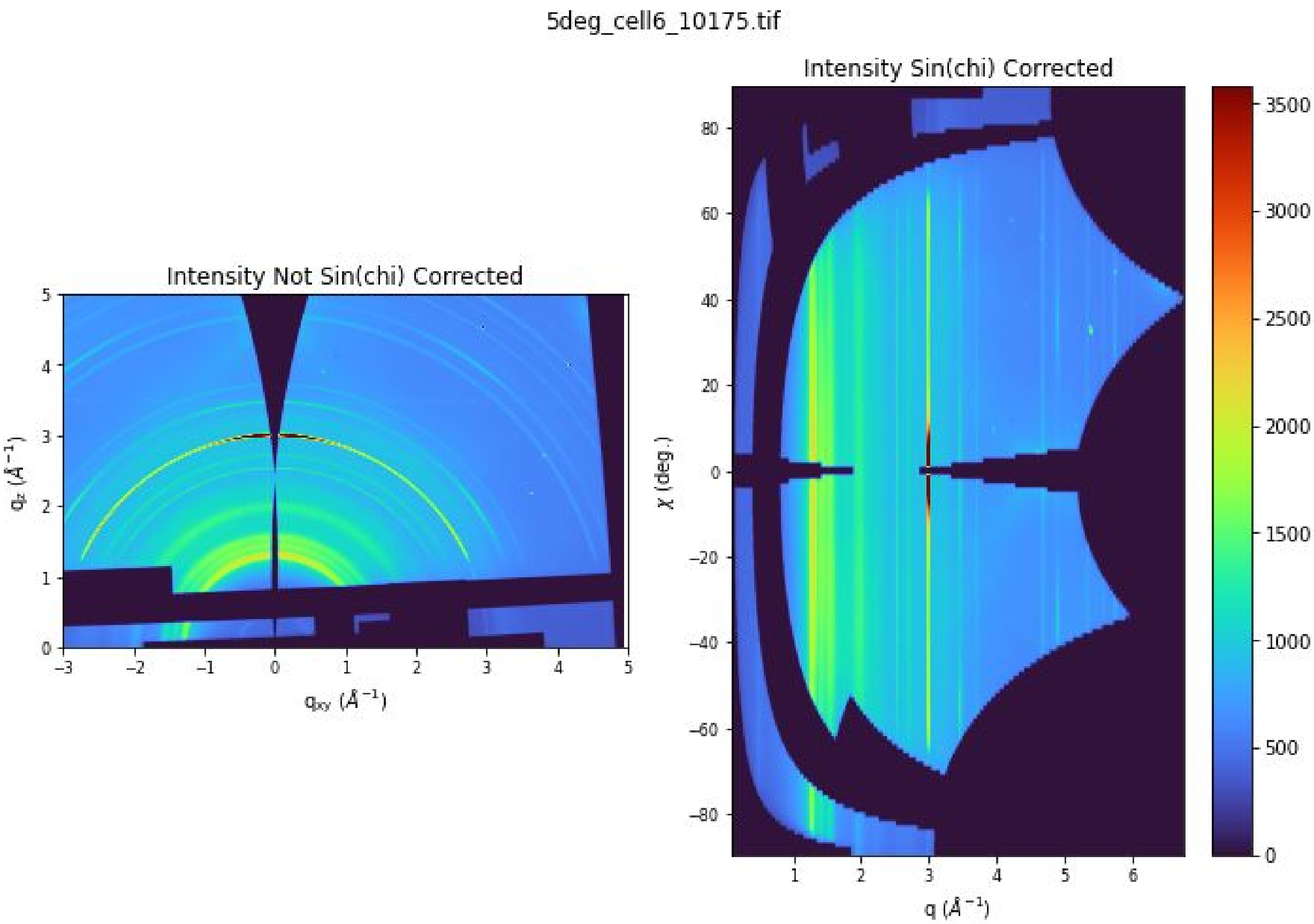

Figure S2. Example of detector image with wedge correction. There is no texturing at zinc peaks, but the copper film is Cu(111) oriented, shown as the bright red peak.

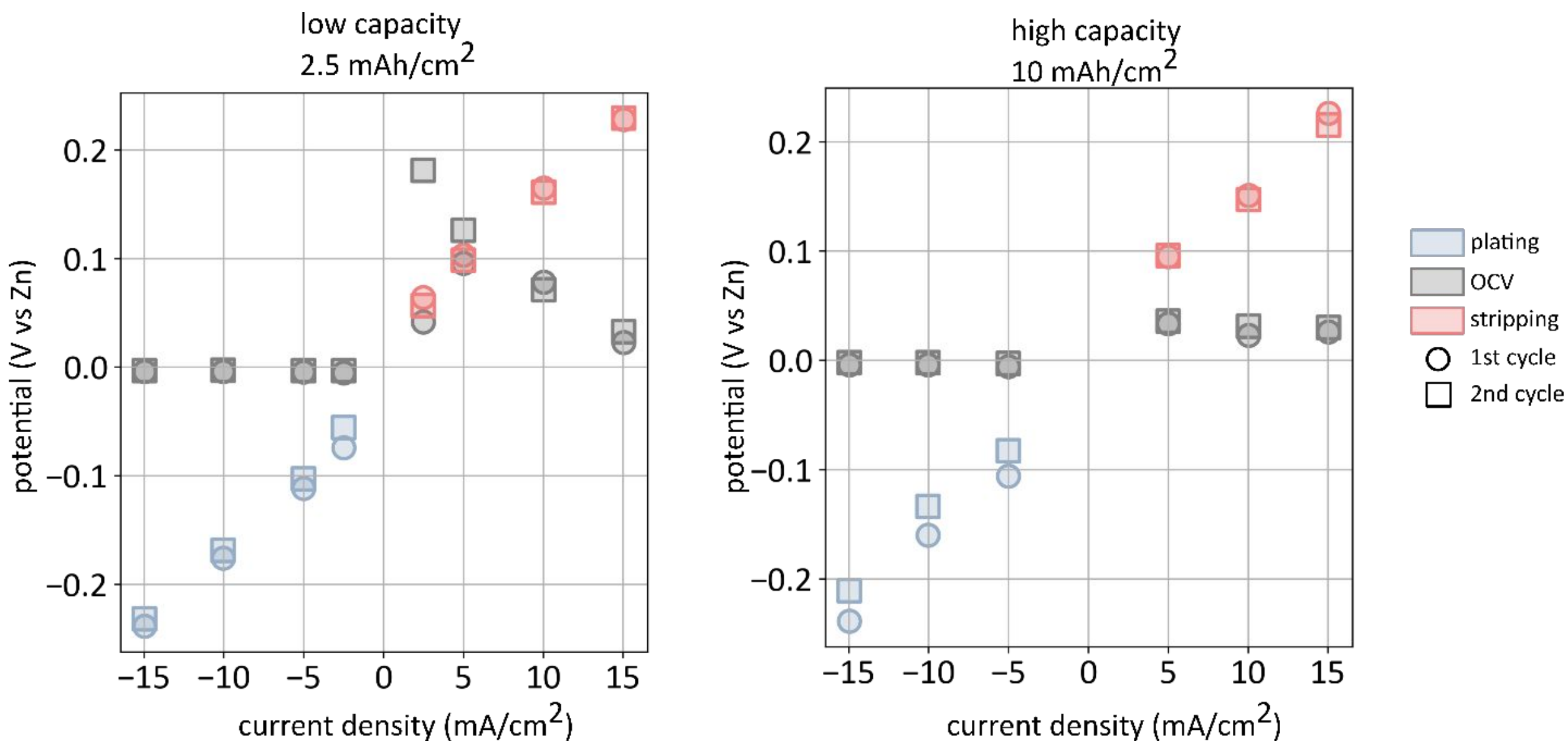

Figure S4. potentials for each cell as a function of current density for plating, stripping, and OCV during the 1st and 2nd cycles.

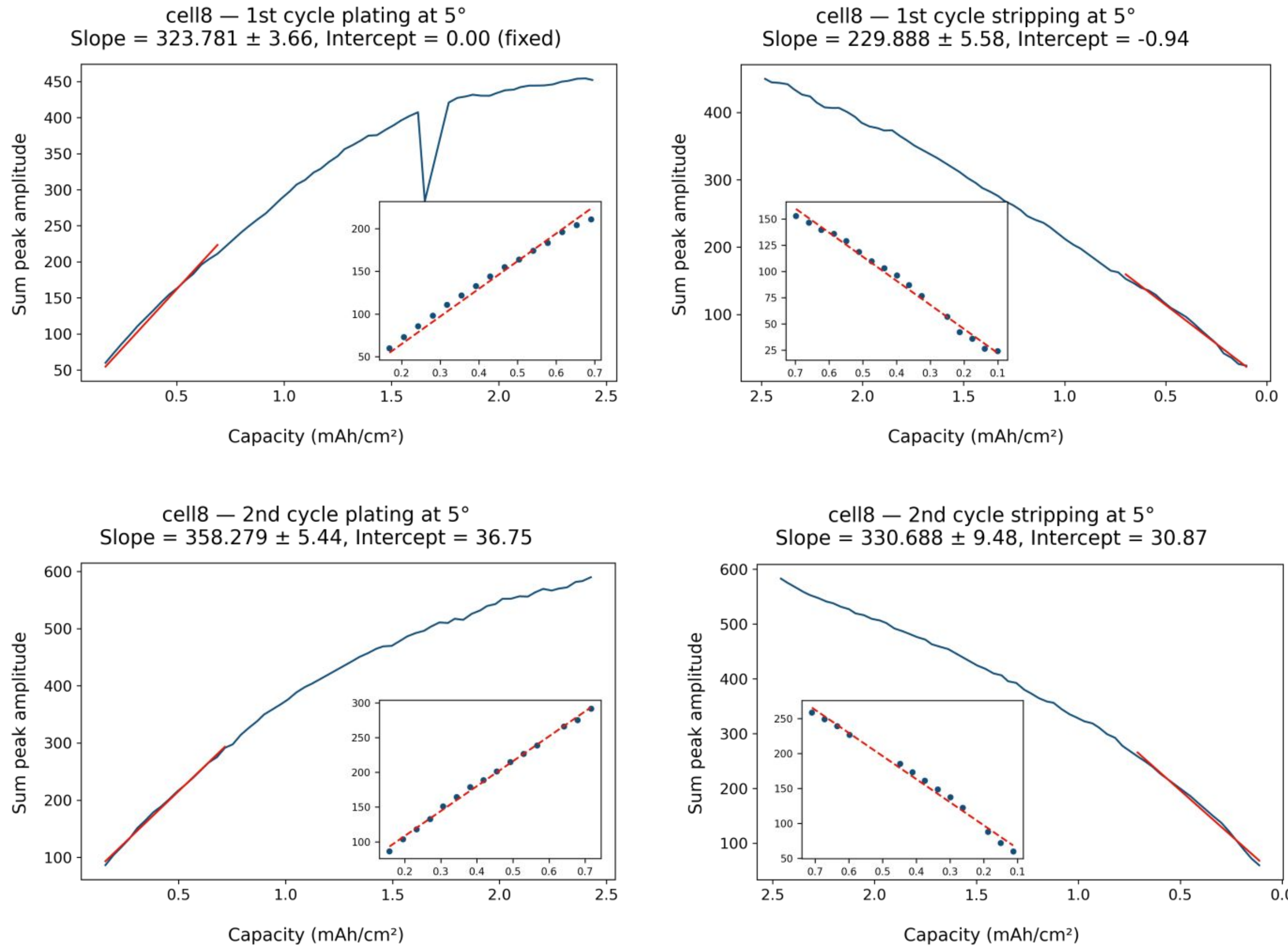

Figure S5. Peak Zn002 fit area at current density 2.5 mA/cm² and capacity 2.5 mAh/cm². Linear fits shown are constrained with intercept = 0.

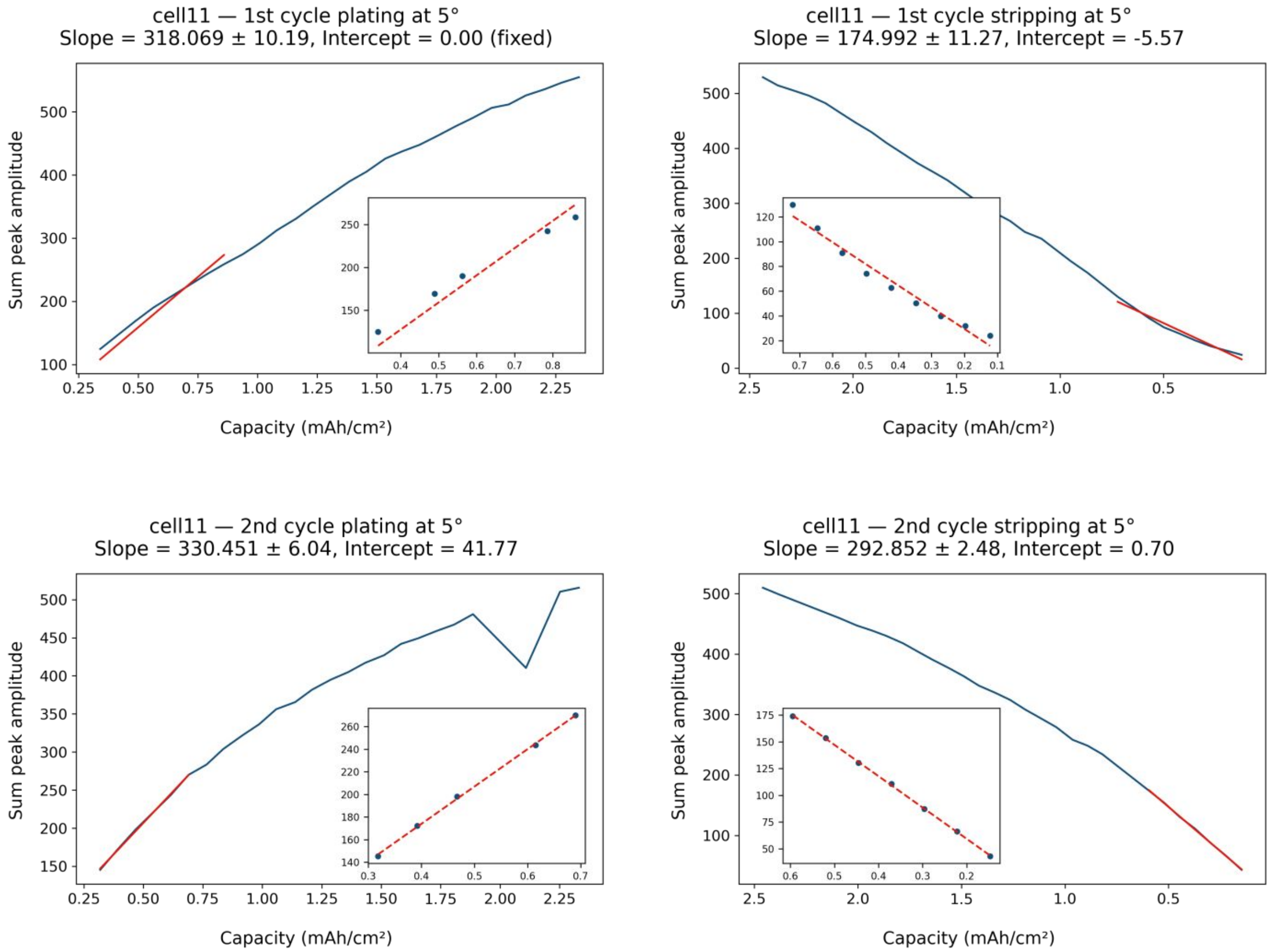

Figure S6. Peak Zn002 fit area at current density 5 mA/cm² and capacity 2.5 mAh/cm². Linear fits shown are constrained with intercept = 0.

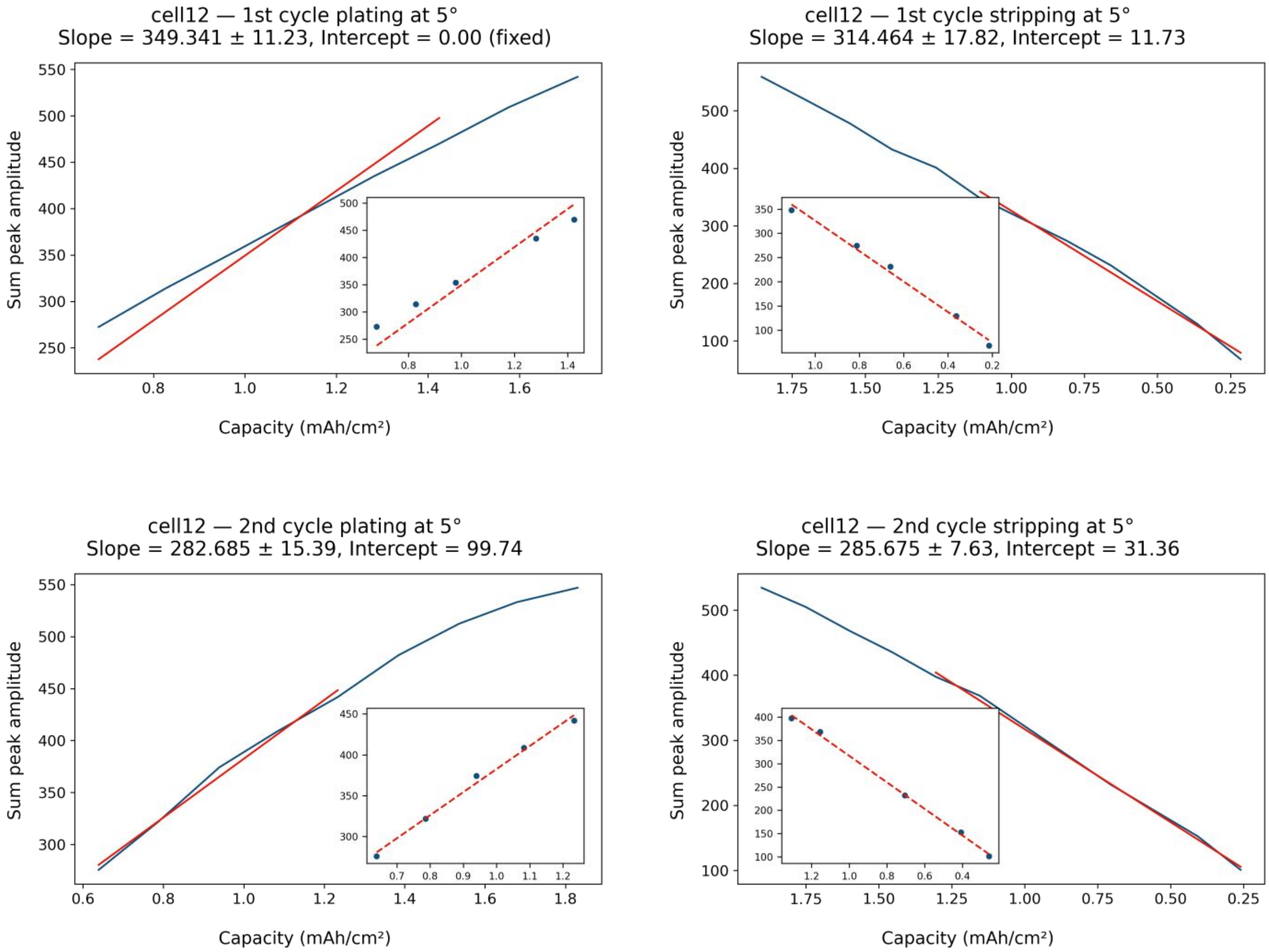

Figure S7. Peak Zn002 fit area at current density 10 mA/cm² and capacity 2 mAh/cm². Linear fits shown are constrained with intercept = 0.

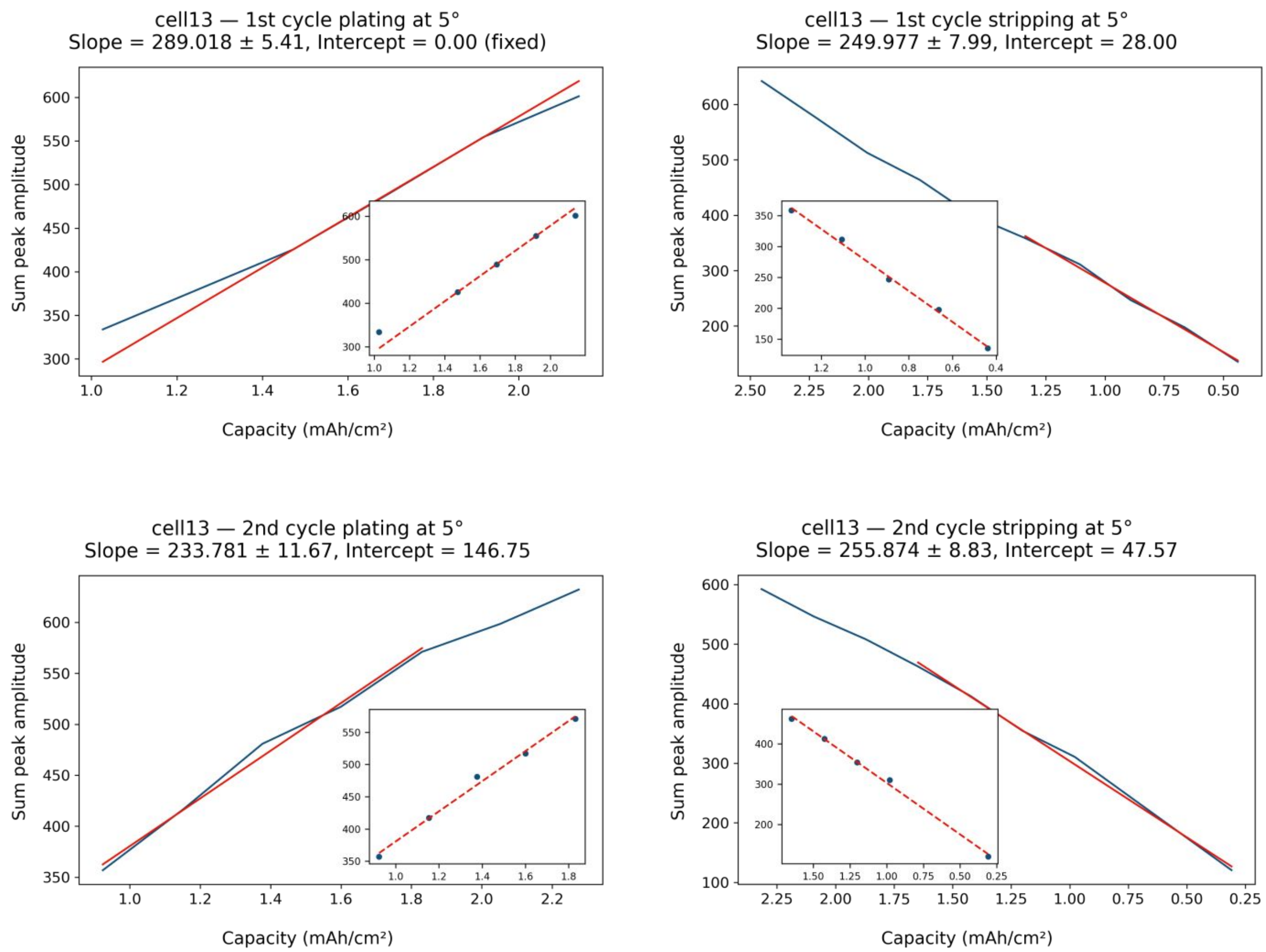

Figure S8. Peak Zn002 fit area at current density 15 mA/cm² and capacity 2.5 mAh/cm². Linear fits shown are constrained with intercept = 0.

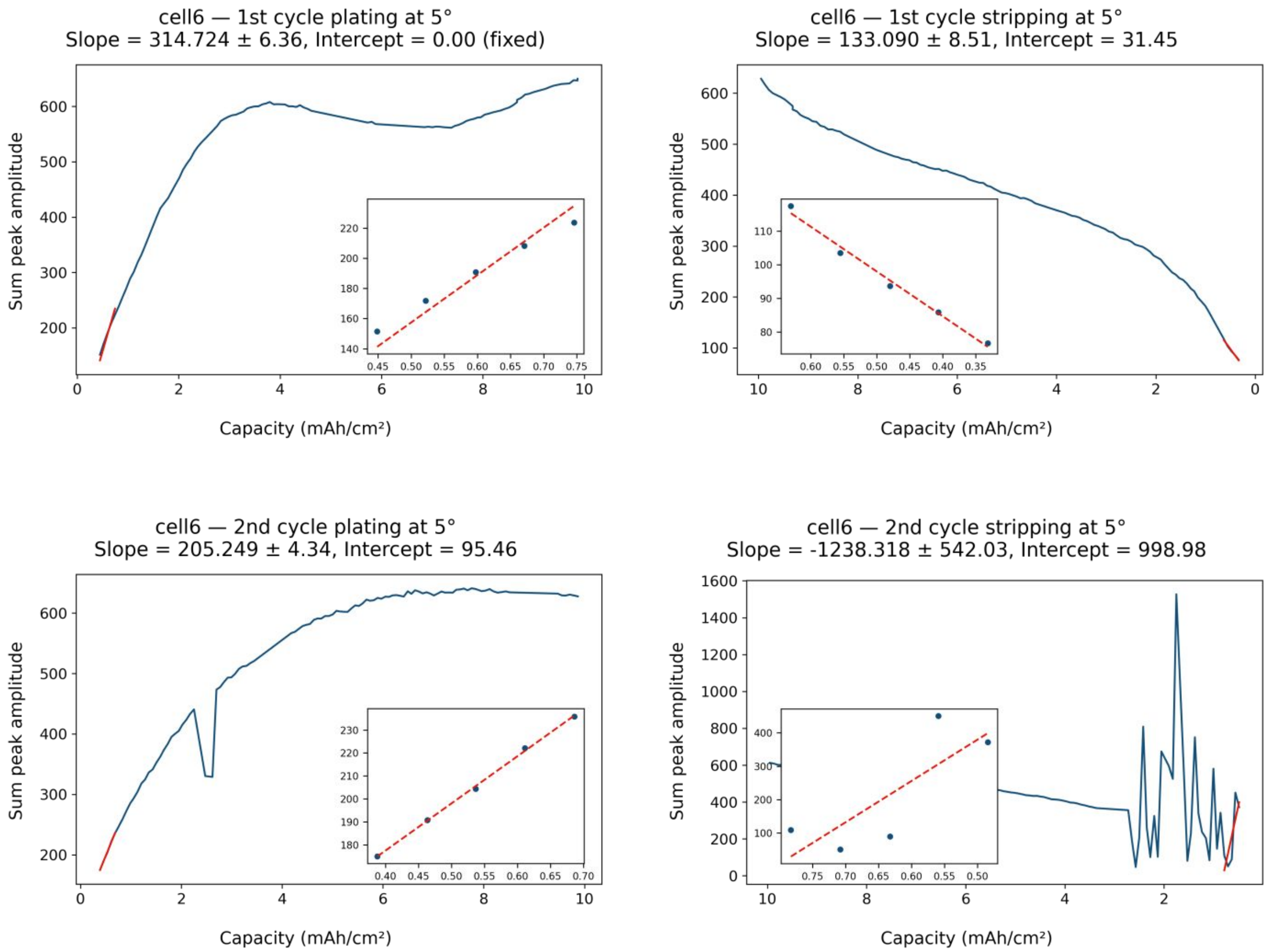

Figure S9. Peak Zn002 fit area at current density 5 mA/cm² and capacity 10 mAh/cm². Linear fits shown are constrained with intercept = 0.

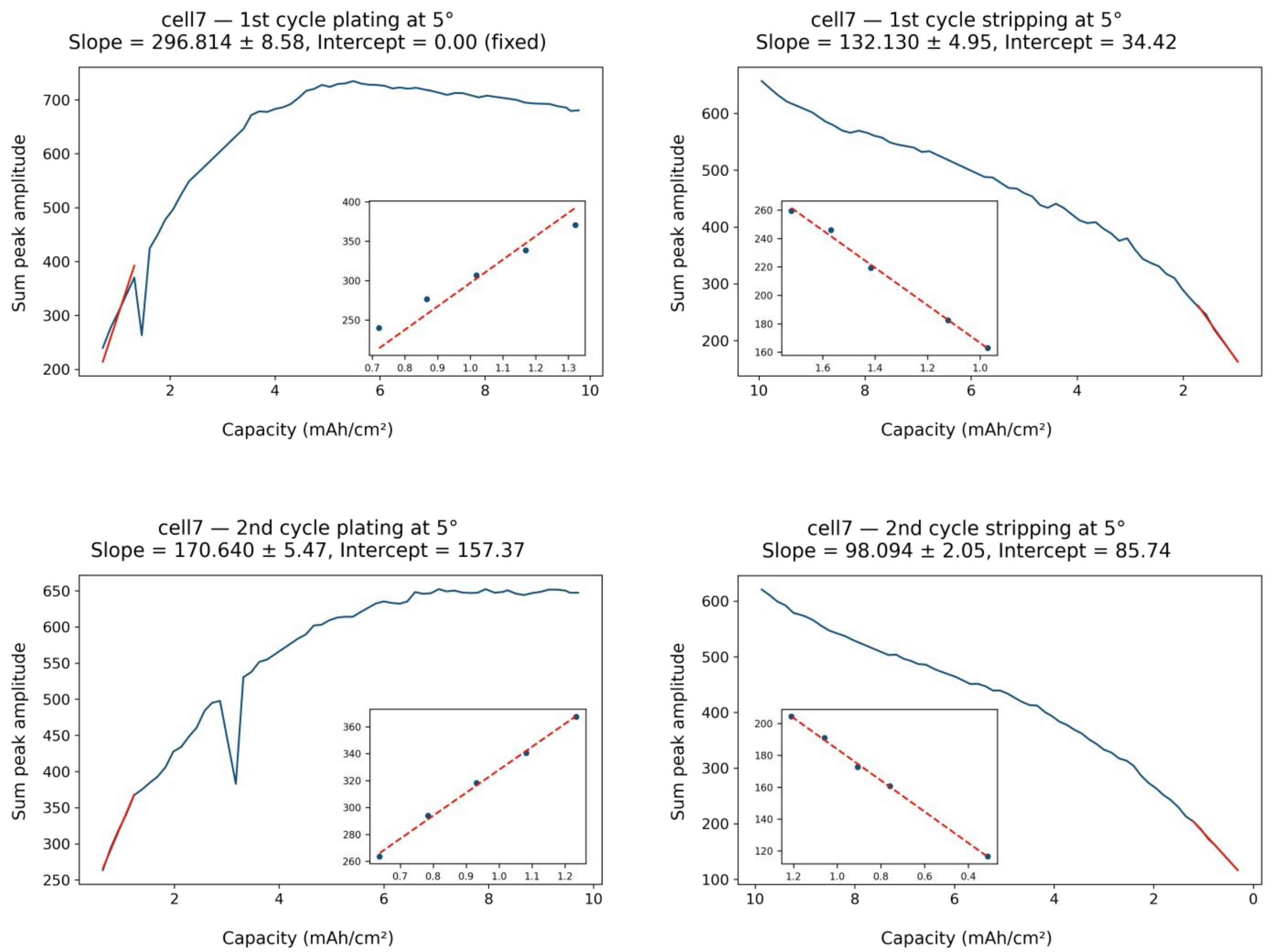

Figure S10. Peak Zn002 fit area at current density 10 mA/cm² and capacity 10 mAh/cm². Linear fits shown are constrained with intercept = 0.

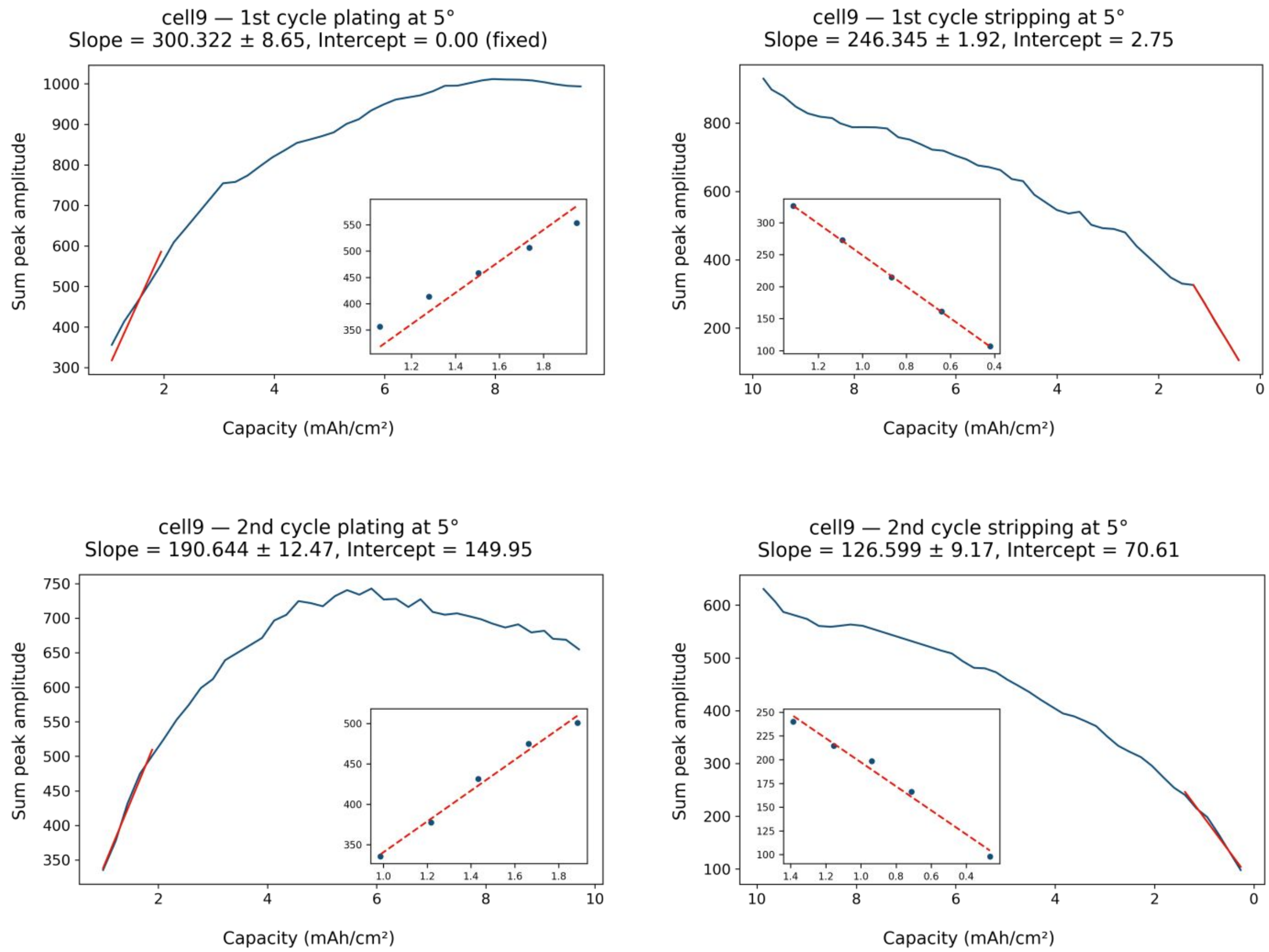

Figure S11. Peak Zn002 fit area at current density 15 mA/cm² and capacity 10 mAh/cm². Linear fits shown are constrained with intercept = 0.

Section S1. Geometric Estimate of X-ray Sampling of Edge-Enriched Regions

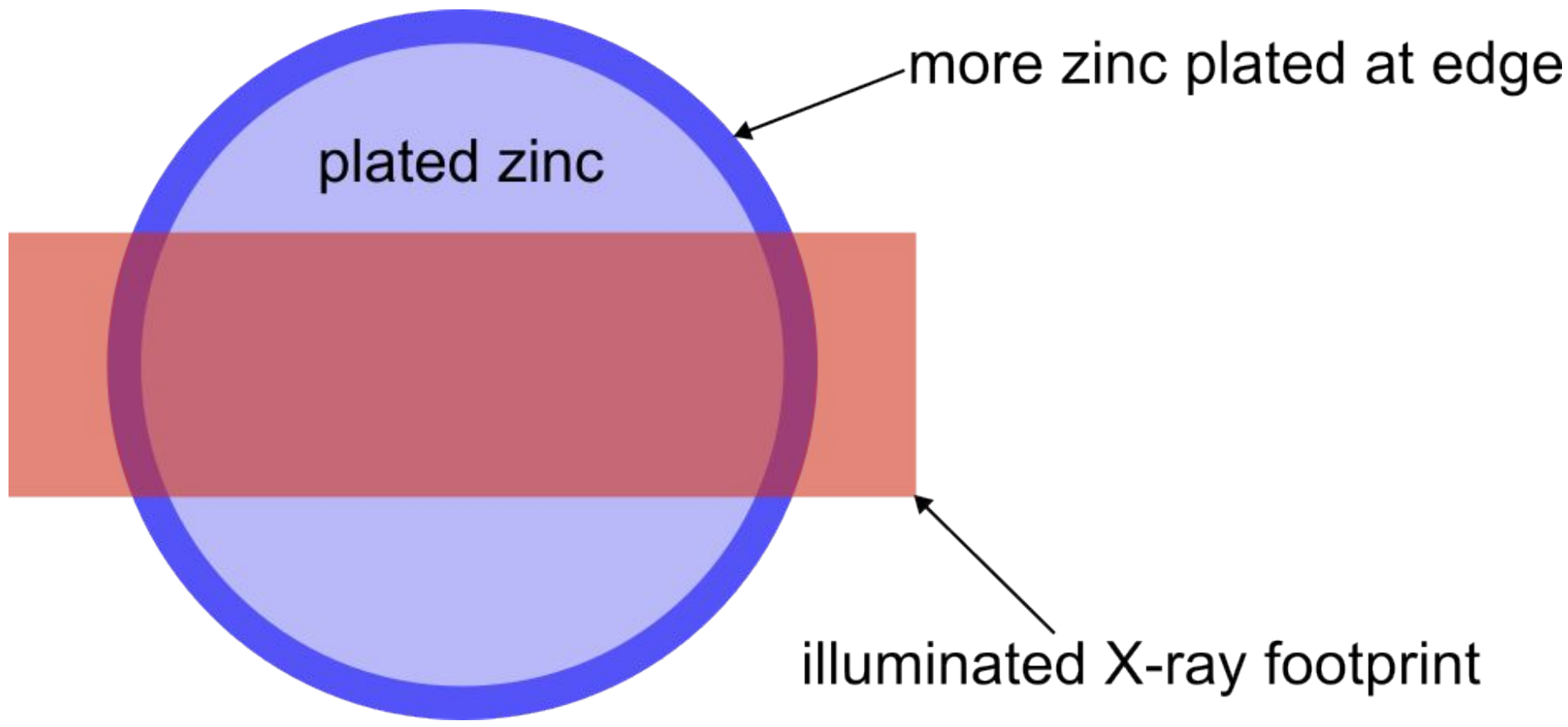

Figure S12. Schematic of how edge plating of zinc is represented in the X-ray footprint.

To evaluate the extent to which the X-ray beam samples Zn deposition at the edges, we estimate the fraction of the active area and edge-enriched regions intersected by the beam. The active area is approximated as a circle of radius R, while the X-ray beam is approximated as a rectangular strip of width w passing through the center of the circle.

The area of the circular region intersected by the beam, the X-ray footprint, is given by:

$$A_{FP}(R) = 2\left[b\sqrt{R^2 - b^2} + R^2 \sin^{-1}\left(\frac{b}{R}\right)\right]$$

where R is the active area radius, b=X-ray width/2, and $A_{total} = \pi R^2$.

So, the fraction of the illuminated X-ray area is:

$$f_{total} = \frac{A_{FP}(R)}{\pi R^2}$$

We can estimate the area of the total area of the edge enriched region will be:

$$A_{edge} = \pi(R^2 - r^2)$$

Where A_{edge} is the area of zinc plated at the edge of the active area, R is the total active area radius, $r = R - \delta$, and δ is the width of the edge-enriched region. Therefore, the portion of the edge region intersected by the beam footprint is:

$$A_{edge,FP} = A_{FP}(R) - A_{FP}(r)$$

And the fraction of the edge region in the total X-ray footprint is:

$$f_{edge} = \frac{A_{FP}(R) - A_{FP}(r)}{\pi(R^2 - r^2)}$$

Our system has a 4 mm diameter (R = 2 mm), a 1 mm wide X-ray beam (b = 0.5 mm), so the beam samples:

$$A_{FP}(R) \approx 3.91\, mm^2$$

Out of a total active area:

$$\pi R^2 \approx 12.57\, mm^2$$

The fraction of active area in the X-ray footprint is:

$$f_{total} \approx 0.31$$

For a 0.127 mm edge region, estimated from SEM images, the total area of the edge region is:

$$A_{edge} \approx 1.55\, mm^2$$

The portion inside of the X-ray footprint is:

$$A_{edge,FP} \approx 0.21\, mm^2$$

Which corresponds to a fraction of the edge area in the X-ray footprint of:

$$f_{edge} \approx 0.135$$

This analysis shows that while the X-ray beam samples ~31% of the total active area, it samples only ~13% of a thin edge region. Therefore, if Zn deposition is enhanced near the perimeter, the measured diffraction signal will underrepresent the total amount of Zn present in the system.

Section S2. Estimation of dead Zn fraction from diffraction peak area

We can estimate the percent of dead zinc by extrapolating the slopes from the low capacity region, assuming the plating rate is constant. With this assumption, the amount of total plated zinc (I_plated) is proportional to:

$$I_{plated} \approx m_{plate} Q_{plated}$$

Where m_{plate} is the initial slope from Figures S5-11, and Q_{plated} is the plated capacity in mAh/cm^2. From there, we estimate the fraction of dead zinc left from the plated zinc:

$$dead\ zinc\ fraction\ = \frac{I_{dead\ Zn}}{I_{plated}}$$

Where $I_{dead\ Zn}$ is the average Zn peak area during the OCV period after stripping, as seen in Figures 4b,d and 6b,d. The resulting values are shown in Table S2:

Table S2. Values used to estimate the fraction of dead Zn from Zn(002) peak area. I is the integrated peak area in arbitrary units. The estimated plated Zn peak area was calculated from the initial linear plating slope multiplied by the plated capacity (equations above). The dead Zn peak area was taken as the average Zn(002) peak area during the OCV period after stripping. Uncertainties in % dead Zn were propagated from the dead Zn peak area and total plated Zn using standard error propagation. The dead Zn uncertainty was estimated from the interquartile range during the 10 minute OCV, and the total plated Zn uncertainty was derived from the slope fitting error.

Current Density (mA/cm²)	Capacity (mAh/cm²)	Cycle	Slope (I / mAh/cm²)	Total Plated Zn (I)	Dead Zn Peak Area (I)	Dead Zn %
2.5	2.5	1st	323 ± 4	809 ± 9	28 (+1.3 / -0.8)	3.5 (+0.2 / -0.1)
2.5	2.5	2nd	358 ± 5	895 ± 14	62 (+0.6 / -0.4)	6.9 (+0.1 / -0.1)
5	2.5	1st	318 ± 2	795 ± 6	13 (+3.2 / -0.9)	1.6 (+0.4 / -0.1)
5	2.5	2nd	330 ± 6	826 ± 15	17 (+2.9 / -1.6)	2.1 (+0.4 / -0.2)
10	2	1st	349 ± 37	698 ± 74	18 (+2.2 / -0.5)	2.6 (+0.4 / -0.3)
10	2	2nd	282 ± 15	565 ± 31	21 (+1.8 / -1.2)	3.7 (+0.4 / -0.3)
15	2.5	1st	289 ± 8	722 ± 20	27 (+0.7 / -2.9)	3.7 (+0.1 / -0.4)
15	2.5	2nd	233 ± 12	584 ± 29	21 (+8.6 / -7.1)	3.6 (+1.5 / -1.2)
5	10	1st	314 ± 2	3147 ± 24	68 (+0.4 / -0.2)	2.2 (+0.0 / -0.0)
5	10	2nd	205 ± 4	2052 ± 43	99 (+2.2 / -34.8)	4.8 (+0.1 / -1.7)
10	10	1st	296 ± 37	2968 ± 371	109 (+1.7 / -1.7)	3.7 (+0.5 / -0.5)
10	10	2nd	170 ± 5	1706 ± 55	98 (+1.3 / -1.4)	5.7 (+0.2 / -0.2)
15	10	1st	300 ± 8	3003 ± 80	61 (+2.5 / -4.1)	2.0 (+0.1 / -0.1)
15	10	2nd	190 ± 12	1906 ± 125	54 (+2.7 / -5.7)	2.8 (+0.2 / -0.4)

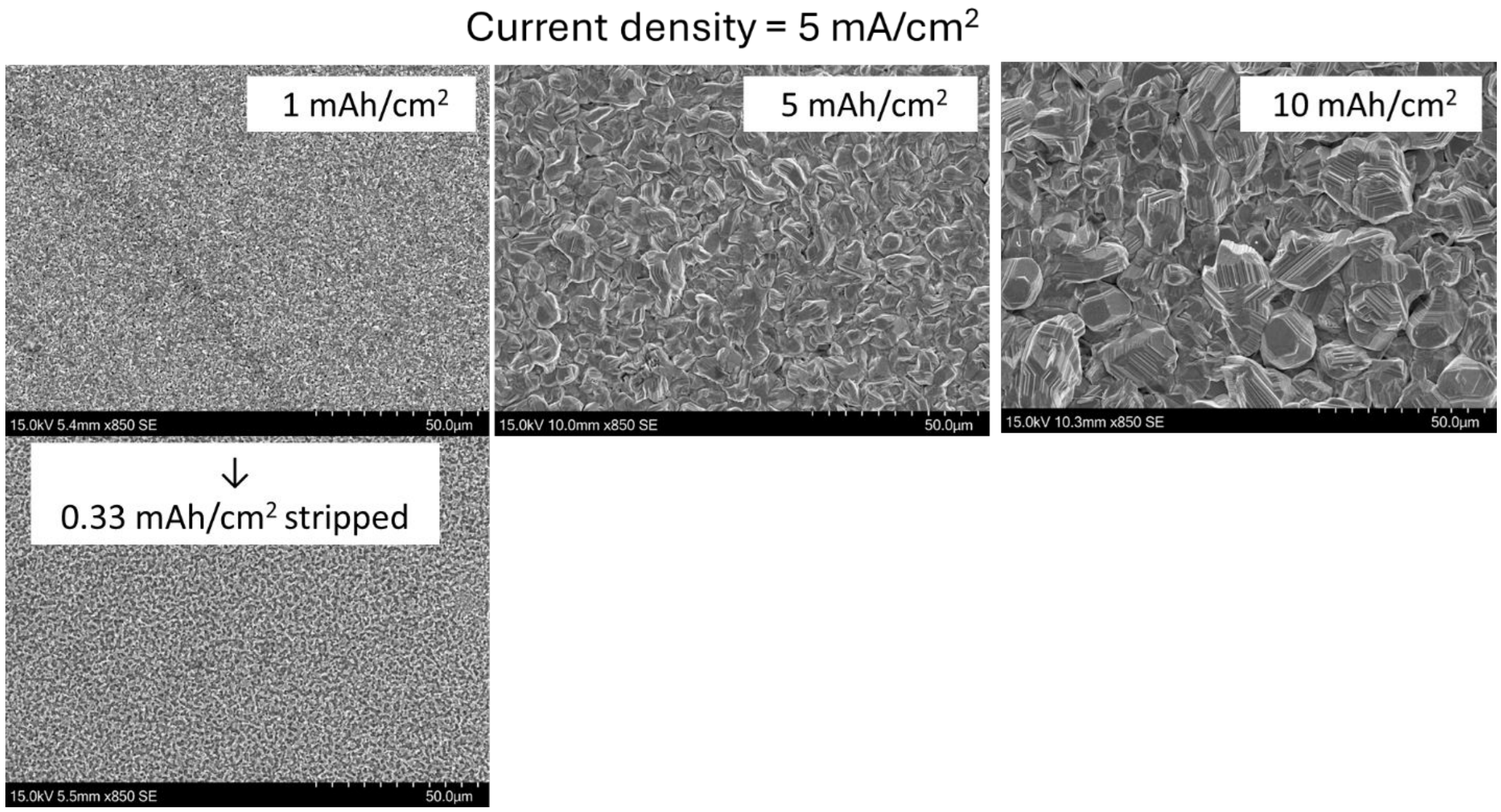

Figure S12. SEM images of plated zinc. Partial stripping at 0.33 mAh/cm² results in a porous morphology. Additionally, zinc crystallite size increases with plating capacity.
